# Evidence of the factors that influence the utilisation of Kangaroo Mother Care by parents with low-birth-weight infants in low- and middle-income countries (LMICs): a scoping review protocol

**DOI:** 10.1186/s13643-018-0714-9

**Published:** 2018-04-05

**Authors:** Christina T. Mathias, Solange Mianda, Themba G. Ginindza

**Affiliations:** Discipline of Public Health Medicine, School of Nursing and Public Health, University of KwaZulu-Natal, Durban, South Africa

**Keywords:** Barriers, Challenges, Facilitating factors, Kangaroo Mother Care utilisation, Low-birth-weight infants, LMICs, Parents

## Abstract

**Background:**

The Sustainable Development Goal (SDG) 3 emphasises on reducing neonatal deaths caused by low birth weight (LBW) complications by the implementation and utilisation of Kangaroo Mother Care (KMC) in low- and middle-income countries (LMICs). Despite the empirical evidence of KMC optimising low-birth-weight infants’ (LBWIs’) survival, its advantages and the LMICs implementing the service, studies have shown that LBW infant deaths occurring in LMICs are largely contributing to global child mortality. The aim of this scoping review is to map out the literature on barriers, challenges and facilitators of KMC utilisation by parents with LBWIs.

**Methods and analysis:**

This scoping review will use Endnote X7 reference management software to manage articles. The review search strategy will use SCIELO and LILACS databases. Other databases will be used via EBSCOHost search engine and these are Academic search complete, CINAHL with full text, Education source, Health source: Nursing/Academic Edition, Medline with full text and Medline. We will also use Google Scholar, JSTOR, Open grey search engines and reference lists. A two-phase search mapping out process will be done. In phase 1, one reviewer will perform the title screening and removal of duplicates. Two reviewers will do a parallel abstract screening according to eligibility criteria. Phase 2 will involve the reading of full articles and exclusion of articles, in accordance with the eligibility criteria. Data extraction from the articles will be done by two reviewers independently and parallel to the data extraction form.

The data quality assessment of the eligible studies will be done using the Mixed Method Appraisal Tool (MMAT). The extraction of the synthesised results and thematic content analysis of the studies will be done by NVIVO version 10.

**Discussion:**

We expect to find studies on barriers, challenges and facilitating factors of KMC utilisation by parents with LBWIs in LMICs. The review outcomes will guide future research and practice and inform policy. The findings will be disseminated in print, electronic and conference presentations related to maternal child and neonatal health.

**Electronic supplementary material:**

The online version of this article (10.1186/s13643-018-0714-9) contains supplementary material, which is available to authorized users.

## Background

### Global prevalence and impact of LBWI on neonatal mortality

Kangaroo Mother Care (KMC), a skin-to-skin contact between the medically stable low-birth-weight infant (LBWI), weighing less than 2500 g, and the parent’s bare chest [1, 2], has proven to reduce LBWI mortality by almost 50% [3–6]. Currently, low birth weight (LBW) mortality is a global leading cause of neonatal mortality contributing to 60–80% of neonatal deaths [7, 8]. Globally, an estimated 20 million low-birth-weight infants (LBWIs) are born annually, with low- and middle-income countries (LMICs) accounting for 18 million births [9]. LMICs bear a higher burden of LBWI outcomes due to the high prevalence of LBWI deaths as compared to high-income countries [3]. Each year, more than 50% of the LBWIs that are born in LMICs do not survive compared to their counterparts born in high-income countries [7].

### Management of LBWIs in LMICs and its outcomes

The high LBWI deaths in LMICs are due to low economic income levels, poverty, poor health-seeking behaviour and weak health systems’ links [7, 10]. As such in LMICs, the management of LBWI complications has been more of Kangaroo Mother Care as compared to conventional/incubator care, since around 1978 [4, 10–13]. LMICs strive to improve neonatal health through the implementation of KMC, among other interventions [2, 14], for instance, incorporating KMC package and guidelines in medical and nursing college syllabi, in-service training and existing national health care initiatives [15–17]. However, although by 2015, 62 countries achieved the three-quarter child mortality reduction target, LBWI mortality contributed largely to child mortality deaths despite the LMICs implementing KMC [8, 18, 19]. Therefore, it is evident that KMC utilisation in LMICs is a challenge as LBWI mortality in LMICs still contributes largely to global neonatal and child mortality [3, 8, 10, 12, 18].

### Problem statement and aim of the study

The measure of success of KMC not only depends on KMC implementation but also on the utilisation of the service by the beneficiaries, parents with LBWIs [20, 21]. As such, the success of service utilisation largely depends on the utilisation determinants, absence of challenges and barriers, perceived quality of care, cost of care, supportive factors, cultural factors, health system factors and provider factors [20]. There are many studies focusing on KMC service delivery and healthcare providers; however, not many studies have focused on factors that influence KMC utilisation by parents with LBWIs [22]. World Health Organization (WHO) emphasises on employing strategies that will facilitate attaining the Sustainable Development Goal (SDG) 3, which aims at reducing newborn deaths to 12 neonatal deaths per 1000 live births per country [8, 10, 18]. In order to enhance achieving SDG 3, this scoping review, therefore, aims at mapping out existing literature on the factors influencing KMC utilisation by parents with LBWIs in LMICs. The objective of the study will be to identify and describe the factors influencing KMC utilisation by parents with LBWI in LMICs.

### Significance of the study

This study will facilitate the identification of strategies/recommendations to inform policy development and/or update and inform future research and practice, by employing approaches to facilitate the uptake of KMC by parents with LBWIs.

## Methodology

### Scoping review

We plan to conduct a scoping of studies on barriers, challenges and facilitating factors of KMC utilisation by parents with LBWIs. The scoping review will facilitate the mapping out of new concepts, evidence-based knowledge and identified knowledge gaps [23]. The proposed study will adopt the framework by Arksey and O’Malley [24]. In summary, the framework involves the following:

i. Identifying the research question
ii. Identifying relevant studies
iii. Study selection
iv. Charting the data
v. Collating, summarising and reporting the results.

#### Identifying the research question

The research question is What is the documented evidence of the factors that influence the utilisation of KMC by parents with LBWI in LMICs? The research sub-questions are:

1. What are the facilitating factors of KMC utilisation by parents with LBWIs in LMICs?
2. What are the barriers and challenges to KMC utilisation by parents with LBWIs in LMICs?
3. What are the experiences of KMC utilisation by parents with LBWIs in LMICs?

##### Eligibility of research question

The amended *SPIDER (Sample, Phenomenon of Interest, Design, Evaluation and Research type)* framework will be used to determine the eligibility of the research question (see Table 1).

**Table 1:** Framework determining eligibility of research question

Criteria	Determinant
Sample	Parents/guardian of LBWIs utilising KMC
Phenomenon of interest	Kangaroo Mother Care
Design	Randomised control clinical trials; non-randomised experiments; survey; cross-sectional, case control and cohort studies
Evaluation	Barriers, challenges, bottlenecks, enablers, experiences and facilitating factors to KMC utilisation
Research type	Qualitative, quantitative and mixed method

#### Identifying relevant studies

The scoping review will include qualitative, quantitative and mixed method primary research articles published in peer-reviewed journals and grey literature that address the research question. The review will include the following study designs: randomised control clinical trials, non-randomised experiments, survey, cross-sectional study designs, case control and cohort studies. The electronic databases that will be used to search for relevant articles will include Academic search complete, CINAHL with full text, Education source, Health source: Nursing/Academic Edition, Medline with full text and Medline. All these electronic databases will be accessed via EBSCOHost search engine. We will also search for studies from SCIELO and LILACS databases. Google Scholar search engine, JSTOR search engine, Open Grey search engine, ‘the cited by’ and reference lists will also be used to search for the relevant literature. Articles written only in English and those that can automatically be translated in English will be reviewed. The LMICs have been implementing KMC since it was introduced in 1978 by Ray; however, we will only include studies published between 1990 and 2017. The year 1990 was marked as the baseline for the United Nations development goals, as such that will be our starting point.

The search terms of this scoping review have originated from indexed subject headings, keywords of relevant studies, terms from this scoping review protocol that recurred repetitively and the Medical Subject Headings (MeSH) terms. The string/Boolean search terms for this review will include kangaroo mother care OR kangaroo care OR skin to skin OR kangaroo-mother care method OR skin to skin contact AND parents OR mother OR father OR family caregivers AND low-birth-weight infants OR preterm infants OR premature infants OR very low birth weight infants AND utilization OR uptake OR compliance AND facilitators OR enablers OR motivators OR experience OR perception OR attitudes. The identified studies will be screened using eligibility criteria.

#### Study selection

The following criteria will guide the selection of studies.

##### Eligibility criteria

Inclusion criteria

Studies meeting the following elements will be included in the study:

- ❖ Studies written in English and in other languages with English version
- ❖ Studies aiming at factors that influence the utilisation of KMC by parents of LBWIs in LMICs.
- ❖ Studies focusing on experience/views/perception of parents with LBWIs on the utilisation of KMC in LMICs.
- ❖ Studies with the above criteria and published between 1990 and 2017

Exclusion criteria

Studies with the following elements will be excluded from the study.

- ❖ Studies written in other languages aside English; without English version
- ❖ Studies with the above inclusion criteria but published before 1990
- ❖ Studies with the above inclusion criteria but focusing on high-income countries
- ❖ Studies with the above inclusion criteria but with incubator care as the phenomenon of interest whether in LMICs or high-income countries

##### Study selection procedure

The selection of studies in this scoping review will involve two phases, as follows:

Phase 1

In phase 1, one reviewer will perform title screening from the proposed databases, by examining the relevance of the study titles to the proposed research purpose. The identified articles will be imported to Endnote X7 reference management software, where duplicates will be removed. Table 2 will report the record of the number of articles identified on the electronic database.

**Table 2:** Electronic search record

Keyword search	Database used	Number of studies retrieved
ᅟ	ᅟ	ᅟ

The Endnote X7 library will then be shared with the two reviewers, who in parallel will independently screen the article abstracts according to the eligibility criteria. The full text of the eligible articles will be searched and kept in the EndNote X7 library. The reviewers will consult the University of KwaZulu-Natal Librarian to assist with the articles that will not have full text.

Phase 2

In phase 2, two reviewers will independently perform a parallel full article screening, following the eligibility criteria, and exclusion of articles with reasons. Data extraction will be performed on the eligible articles identified during the full article screening. Two reviewers will do the data extraction in parallel and independently, according to the data charting form as presented in Table 3. Notes will be shared between reviewers during abstract screening and full article screening. Where disagreements arise due to inclusion or exclusion of articles, the articles will be sent to the third reviewer for reassessment and consideration.

**Table 3:** Data charting form

Author and date		
Title of the study		
Aim of the study/ research question		
Population ❖ Sample size ❖ Characteristics of participants • % and number of males • % and number of women • Age/average		
Intervention		
Study design		
Recruitment setting		
Sampling strategy		
Data collection (methodology)		
Data analysis		
Outcome of the study/results		
Conclusion of the study		
Significant findings		
Comments		

**Fig. 1:**
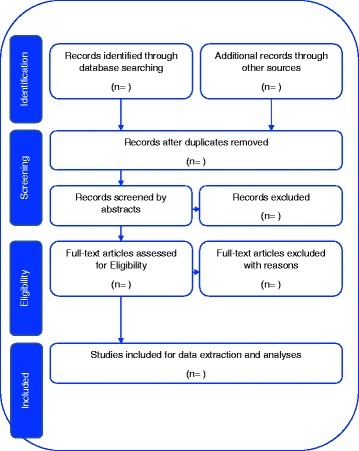
Study selection process

Throughout the selection of eligible articles, the reviewer will keep account of the number of the articles imported to the Endnote X7 and the number of duplicates removed and keep account of the number of eligible articles for the abstract screening. During the abstract screening, the reviewers will take note of the number of excluded articles, indicating the reason for exclusion, and keep the number of articles eligible for full article screening. Reviewers for full article screening will also keep account of the number of articles excluded with reasons. The summary of the study selection process is shown in Fig. 1. The PRISMA-P checklist has been used to guide writing this scoping review protocol, as shown in Additional file 1. However, we will not assess the risk-of-bias assessment, which includes study-level bias, review-level bias and publication bias, as they are irrelevant to the nature of the scoping review [25].

The search strategy will be piloted to determine the validity and reliability of the criteria of the study selection. Table 4 in the Appendix narrates the pilot search results done on Medline via EBSCOHost search engine using the MeSH terms.

Charting the data

We plan to use the elements included in the data charting form (see Table 3) to facilitate the standardised data extraction. The elements in the data charting form will be regularly updated, in order to address the research question.

#### Collating, summarising and reporting the results

The study outcomes that will guide data extraction will include challenges of KMC utilisation, barriers of KMC utilisation and facilitating factors of KMC utilisation. The thematic content analysis will be used to code arising themes and analyse the narrative account of the extracted data. The extracted data will be coded using NVIVO software version 10 [26]. Three stages will be used to guide collating, summarising and reporting of the results. The stages include coding text, developing descriptive themes and generating analytical themes [27]. However, these stages are interrelated in such a way that the free coding of the primary study findings facilitates the organisation of the codes into descriptive themes. The descriptive themes enhance the development of the analytical themes.

##### Stages 1 and 2: Coding text and developing descriptive themes

In this review, two reviewers will independently and in parallel do the line-by-line coding of the primary study findings, in relation to the context and meaning. However, the coding of the study findings will not strictly depend on the research question, as there might be few studies addressing the review question directly [27]. The reviewers will then categorise the initial codes into major groups, depending on their similarities and differences, then new codes will be assigned to these grouped codes, in order to give a descriptive meaning to the groups. Hence, descriptive themes will be developed. One reviewer will write a draft summary of the descriptive themes, which will be reviewed by the rest of the reviewers and they will agree on the final version of the descriptive themes.

##### Stage 3: Generating analytical themes

Independently and in parallel, the reviewers will deduce the barriers, challenges and facilitating factors of KMC utilisation by parents with LBWIs from the descriptive themes. In this stage, the reviewers will, through narration, analyse the descriptive themes and examine the relationship between themes to the review question. The reviewer will then examine the meaning of the study findings to the review question. The implications of the findings will be considered for intervention development. Through the narrative analysis process, individually, the reviewers will be able to develop analytical themes and proposed interventions. Then, the reviewers will together discuss the review question and the implications in relation to the descriptive themes. During the group discussion, we anticipate that more analytical themes and implications for intervention development will emerge. The process will be repeated until we no longer have emerging analytical themes and implications for intervention development. The reviewers will summarise stage 3 by agreeing and approving the identified analytical themes and implications for intervention development. The implications will form the recommendations of the review. Finally, the results will be reported.

## Quality appraisal

The Mixed Method Appraisal Tool (MMAT) version 2011 will be used to assess the quality of the eligible studies in terms of the appropriateness of the study aim, the study design, methodology, sampling strategy, data collection, data analysis, result presentation, discussion and conclusion.

## Discussion

This scoping review will form part of the study on ‘Utilization of Kangaroo Mother Care in Mangochi district, Southern Malawi’. Mapping out the evidence that exists on barriers, challenges and facilitating factors of KMC utilisation in LMICs will provide evidence-based knowledge gaps, inform future research and enrich the main study’s findings. The barriers and challenges to KMC utilisation that prevent parents with LBW infants from utilising the only low-cost effective measure in LMICs that manages LBW complications and prevents LBWIs’ deaths will be identified. The outcomes of this scoping review will inform future research and identify the evidence-based interventions, which will inform policies and guidelines. This is in order to improve KMC utilisation in LMICs, prevent LBWI deaths and contribute towards the SDG 3 goal of 12 neonatal deaths per 1000 live births per country by 2030 [3, 8]. Although rigorous steps will be followed throughout this review, we anticipate some limitations. Firstly, studies may be omitted from the review if they were not published in the databases searched or if they were not published at all. Secondly, articles may not be accessible if they were published in languages other than English.

### Additional file

Additional file 1: The PRISMA-P 2015 checklist. (DOCX 26 kb)

## Appendix

**Table 4:** Results of the pilot database search

Keyword search	Date of search	Search engine used	Number of retrieved studies
(MM “Kangaroo-Mother Care Method/UT”) AND (MM “Infant, Very Low Birth Weight+”) AND (MM “Infant, Low Birth Weight+/GD”) AND (MM “Infant, Extremely Low Birth Weight/GD”) AND (MM “Mothers/PX”) AND (MM “Infant Care+/MT”) Date of publication: 19900101–20171231 Source types: Academic Journals	13 February 2018	Medline via EBSCOHost	976

## Abbreviation

KMC: Kangaroo Mother Care
LBW: Low birth weight
LBWI: Low-birth-weight infant
LBWIs: Low-birth-weight infants
LMICs: Low- and middle-income countries
MMAT: Mixed Method Appraisal Tool
SDGs: Sustainable Development Goals
SPIDER: Sample, Phenomenon of Interest, Design, Evaluation and Research type
UKZN: University of KwaZulu-Natal
WHO: World Health Organization
